# Manipulation of the Growth Hormone-Insulin-Like Growth Factor (GH-IGF) Axis: A Treatment Strategy to Reverse the Effects of Early Life Developmental Programming

**DOI:** 10.3390/ijms18081729

**Published:** 2017-08-08

**Authors:** Clare M. Reynolds, Jo K. Perry, Mark H. Vickers

**Affiliations:** Liggins Institute, University of Auckland, Auckland 1142, New Zealand; c.reynolds@auckland.ac.nz (C.M.R.); j.perry@auckland.ac.nz (J.K.P.)

**Keywords:** growth hormone, insulin-like growth factor, developmental programming, epigenetics, early life nutrition

## Abstract

Evidence from human clinical, epidemiological, and experimental animal models has clearly highlighted a link between the early life environment and an increased risk for a range of cardiometabolic disorders in later life. In particular, altered maternal nutrition, including both undernutrition and overnutrition, spanning exposure windows that cover the period from preconception through to early infancy, clearly highlight an increased risk for a range of disorders in offspring in later life. This process, preferentially termed “developmental programming” as part of the developmental origins of health and disease (DOHaD) framework, leads to phenotypic outcomes in offspring that closely resemble those of individuals with untreated growth hormone (GH) deficiency, including increased adiposity and cardiovascular disorders. As such, the use of GH as a potential intervention strategy to mitigate the effects of developmental malprogramming has received some attention in the DOHaD field. In particular, experimental animal models have shown that early GH treatment in the setting of poor maternal nutrition can partially rescue the programmed phenotype, albeit in a sex-specific manner. Although the mechanisms remain poorly defined, they include changes to endothelial function, an altered inflammasome, changes in adipogenesis and cardiovascular function, neuroendocrine effects, and changes in the epigenetic regulation of gene expression. Similarly, GH treatment to adult offspring, where an adverse metabolic phenotype is already manifest, has shown efficacy in reversing some of the metabolic disorders arising from a poor early life environment. Components of the GH-insulin-like growth factor (IGF)-IGF binding protein (GH-IGF-IGFBP) system, including insulin-like growth factor 1 (IGF-1), have also shown promise in ameliorating programmed metabolic disorders, potentially acting via epigenetic processes including changes in miRNA profiles and altered DNA methylation. However, as with the use of GH in the clinical setting of short stature and GH-deficiency, the benefits of treatment are also, in some cases, associated with potential unwanted side effects that need to be taken into account before effective translation as an intervention modality in the DOHaD context can be undertaken.

## 1. Introduction

Normal growth requires a suitable nutritional supply to the developing fetus and neonate. Suboptimal nutrition during these early critical windows can affect body growth, and enhances the risk for a range of cardiometabolic diseases in later life, a process preferentially termed the developmental origins of health and disease (DOHaD). The DOHaD framework originated from the early work undertaken by Professor David Barker (the “Barker Hypothesis”) that linked the fetal and early infant environment to lasting effects on the body’s metabolism and increased susceptibility to a range of chronic conditions later in life [1,2,3]. The original observations used geographical studies to show that variations in mortality from coronary heart disease correlated closely with death rates among newborn babies, with most deaths linked to low birth weight. The association between low birthweight and heart disease was subsequently validated in a number of longitudinal studies of both men and women [1]. To develop the hypothesis further, Barker and colleagues examined how fetal undernutrition (UN) at different gestational stages was associated with different birth phenotypes, with each differential adaptation linked with specific changes in concentrations of fetal and placental hormones and different metabolic derangements in later life [3]. This integrated framework proposed that “undernutrition during gestation reprograms the relationship between glucose and insulin and between growth hormone (GH) and insulin-like growth factor (IGF)”, which can lead to permanent changes in the body’s structure and function, and increase the risk for a range of diseases in adulthood [4].

The link between a poor early life environment and an increased risk for growth and metabolic disorders in later in life has now been clearly evidenced via clinical and human epidemiological studies and a vast array of experimental animal paradigms across a range of model species, including rodents, sheep, guinea pigs, and the non-human primate. In particular, poor maternal nutrition, including both UN and overnutrition, can lead to obesity and a range of related cardiometabolic disorders in offspring in later life. This process of developmental programming leads to an offspring phenotype that closely resembles that of growth hormone deficiency (GHD), including sarcopenia, increased adiposity, lipid abnormalities, cognitive deficits, impaired insulin and adipokine sensitivity, and altered cardiac function [5]. However, there is a paucity of data around long-term alterations in GH-IGF axis regulation following early life programming, as most studies have focused on the fetal or early neonatal period. In clinical cases, primarily reported in pregnancies complicated by intrauterine growth restriction (IUGR), circulating glucose, insulin, IGF-1, and IGF-2 concentrations in the umbilical cord are significantly decreased. Experimentally, both maternal UN and maternal obesity are characterised by a reduction in circulating IGF-1 concentrations in offspring at birth [6,7], with IGF-1 being used as a crude proxy for GH status given the logistical issues around the measurement of GH, which requires serial sampling due to a pulsatile secretion profile. However, it needs to be noted that the status of the GH-IGF axis at birth or in the early postnatal life in children born small for gestational age is not a great predictor of later growth with circulating concentrations of GH, IGF-1, IGF binding protein-3 (IGFBP-3) shown to be unrelated to subsequent growth patterns [8,9].

Developmental programming was once considered as an irreversible change in developmental trajectory. However, a number of experimental studies have now shown that programming is indeed reversible via interventions during critical early windows of developmental plasticity [10]. The somatotropic axis is highly sensitive to nutritional supply during the early developmental period [11]. Given the sensitivity of GH to nutritional influences and the observed changes in the GH-IGF axis in both mothers and offspring resultant from poor early life nutrition, intervention strategies using GH or components of the GH-IGF system thus appeared as strong candidates to ameliorate developmental malprogramming. Most evidence to date has been derived from a range of both small and large experimental animal models. Limited clinical evidence in the setting of programming is available, as the use of GH in the clinic is restricted to those with tightly defined short stature and GHD, and there are potential safety concerns around the use of GH and subsequent diabetes/cancer risk [12,13].

In the setting of suboptimal maternal nutrition, work by Donzeau et al. suggested that in cases of fetal growth restriction, the programming of GH sensitivity may represent one of a number of potential (mal)adaptations to an expected suboptimal nutritional environment postnatally, thus acting to restrict the anabolic actions of GH [14]. This fits with the concept of developmental mismatch and “predictive adaptive responses” (PARs) [15], by which cues that are received by the fetus in early life can influence the development of a phenotype that is adapted to the expected postnatal environment. When the predicted and actual environment conditions differ, the mismatch between the individual’s adaptive phenotype and the actual environment in which it exists can have adverse impacts on later health outcomes [15].

Limited data from epidemiological cohorts has provided evidence for persistent alterations in the GH-IGF axis arising from a poor start to life. As an example, in postmenopausal women, childhood exposure to famine during the Dutch Hunger Winter is associated with increased circulating plasma concentrations of IGF-1 and IGFBP-3, with decreases in IGFBP-1 and IGFBP-2 [16]. These data are opposite to those seen under conditions of starvation, and thus may reflect the effect of a persistent maladaptation induced by programming arising due to improved nutrition following the famine period (a possible consequence of inappropriate PARs as detailed above). Of note, these changes in the IGF-IGFBP axis may be linked to the increased risk of cardiovascular disease and breast cancer observed in female offspring exposed to the Dutch famine. However, these changes are associative and no direct causality has been evidenced; elevations in disease risk may reflect aberrations across a number of regulatory systems, and key tissues arise due to famine exposure for which changes in IGF-IGFBPs may represent a surrogate measure [17]. However, there are no data as to the effectiveness of restoring GH in these cohorts, and the data remain observational in nature.

The use of recombinant human GH (hGH) for the treatment of short stature in children only commenced in 1985, and therefore the longer term effects of GH on cardiometabolic health are not well defined. Prior to this, therapy in the form of cadaver-derived GH was linked to cases of Creutzfeld–Jacob disease. Further, the prevalence of small for gestational age (SGA) and short stature in children born SGA who meet entry criteria for GH treatment is very low, with some reporting an estimated prevalence of short stature in those children born SGA who qualified for GH treatment being as low as 0.06% of all cases [18]. In children with GHD, short term GH treatment can lead to impaired insulin sensitivity in the absence of changes in glucose tolerance [19]. Earlier data had suggested that GH treatment in children and adolescents resulted in a marked increase in the incidence of Type 2 diabetes (T2DM), with the T2DM not resolving after the cessation of treatment [20]. However, some studies suggest a positive and long-term influence of GH treatment in children with GHD across a range of outcomes, including β-cell secretory capacity (adaptive response to changes in insulin sensitivity) [21], blood pressure, body composition, bone health, and lipid metabolism [22], and GH therefore appears to be a safe drug as an agent to promote growth in short children born SGA, based on current dosage practices [23,24]. In infants born preterm and SGA, GH administration has been used as a means to stimulate protein synthesis, but no significant effects on linear growth, body composition, and protein or glucose homeostasis were observed. Of note, circulating concentrations of IGF-1 and IGFBP-3 showed normal developmental increases and were not altered with GH treatment: this may reflect a relative insensitivity to GH or GH resistance during the early preterm period [25].

In addition to clinical applications for childhood and adult GHD, recombinant GH has been used as an agent to improve milk yield in animals and humans. Early studies demonstrated that the administration of bovine GH (bGH) improved milk yield in dairy cows, and recombinant bovine GH is still used today in some countries. Studies undertaken in rodents demonstrated that GH was essential for mammary development both in the pubertal phase and during pregnancy [26]. In the clinical setting, hGH therapy to mothers with lactational insufficiency following preterm birth can improve breast milk volumes with parallel increases in IGF-1 and IGFBP-3, but not GH, plasma concentrations [27]. In normal lactating women, a short term administration of GH can increase markers of cellular proliferation but not milk protein gene expression [28]. Maternal obesity in the clinical setting and in experimental models has been linked to lactation failure and increased neonatal mortality [29], but the efficiency of GH treatment in this setting has yet to be examined. It has recently been shown in the GH transgenic goat that GH overexpression in the mammary gland can stimulate tissue development and enhance milk yield by modulating alveolar cell proliferation/branching via the mitogen-activated protein kinase (MAPK) signaling pathway [30].

## 2. Animal Models of Manipulation of the GH-IGF Axis

Most of the work on the use of GH as a strategy to reverse the effects of developmental programming have been undertaken in experimental models of maternal nutritional deficit, including global UN and targeted nutrient restriction [31,32,33]. Of note, the changes in the GH-IGF system in mammalian models of programming studied to date appear relatively conserved.

### 2.1. Rodents

Recent work in a dwarf rat model with developmental GH-IGF-1 deficiency has highlighted that the programming of the GH-IGF axis during critical periods of development can determine cellular DNA repair capacity via alterations in the transcriptional regulation of DNA repair-related genes. This can manifest as changes in cellular resistance, including responsiveness to cell stressors and environmental toxins [34]. Peripubertal GH treatment reverses the resistance phenotype. Given that early lifestyle environmental factors (e.g., nutrition and physical activity) can lead to marked variations in early GH/-IGF-1 concentrations in childhood, further studies are warranted to determine any persistent influences on cellular resistance pathways related to cardiometabolic disease outcomes.

In adult rat offspring of UN mothers, GH treatment can normalise systolic blood pressure and adiposity, but these beneficial changes were accompanied by hyperinsulinemia in offspring, particularly in those fed a post-weaning high fat diet, and an increased heart to body weight ratio reflecting cardiac hypertrophy [31]. In a further model of maternal UN, IGF-1 treatment in adulthood led to a reversal of most of the adverse phenotypic changes in offspring, with the normalisation of fat mass, blood pressure, appetite, and leptin and insulin concentrations [35]. A limitation, however, of these studies is that sex-specific effects were not examined, and as previously shown for other treatment interventions including neonatal leptin, outcomes may be sexually dimorphic in nature [36,37].

It is well established that GH treatment can be associated with adverse side effects (in part related to dosing issues and the duration and timing of GH exposure), including impaired insulin sensitivity and fluid retention. Further, GH treatment does not result in the optimization of final height in those children with idiopathic short stature [38]. Given that a reduction in free fatty acids via pharmacological anti-lipolysis agents can lead to the stimulation of GH secretion in both normal subjects and those with GHD, combination treatment approaches to both mitigate the unwanted side effects of GH treatment and maximize GH efficacy have been examined. In a rodent model of maternal UN, a combination treatment with GH and the lipid lowering agent acipimox resulted in an enhancement of linear growth in both control and maternally undernourished male offspring, and prevented the hyperinsulinemia that resulted from GH treatment alone; these effects of acipimox may be mediated in part by inducing a direct decrease in free fatty acids that are known to inhibit GH responsiveness to a range of pharmacological and physiological stimuli [39,40]. Clinically, it has also been shown that pharmacological anti-lipolysis agents can restore insulin sensitivity during GH exposure [41,42]. Further combinatorial approaches include the clinical use of combination therapy with GH and aromatase inhibitors or GH and gonadotropin-releasing hormone (GnRH) analogs to enhance growth potential [43,44].

Outcomes in the rodent work may also be dependent upon the severity of the UN model used. In the setting of moderate UN (50% of ad libitum), pre-weaning GH treatment appears to resolve most of the adverse outcomes arising in offspring in later life [32,45,46]. However, the administration of GH or IGF-1 to pregnant rat dams under a paradigm of severe global food retraction (70% reduction throughout pregnancy) did not prevent fetal IUGR, nor did it prevent elevations in blood pressure in adult offspring [47]. Further, there is potential for the data derived from animal models to differ depending upon the type of GH used, e.g., non-homologous systems. Studies in rodents to date have primarily used bGH or hGH, which differ in amino acid sequence and function; for example, hGH but not bGH can exert lactogenic effects. Thus, in the rodent, hGH can bind to both somatogenic and lactogenic binding sites, whereas bGH is thought to be specific only to somatogenic binding sites. As an example, phenotypic outcomes arising from the induced expression of hGH and bGH in transgenic mice are different, and in some cases opposing [48].

Although the peripheral effects of GH/IGF treatment have been well-characterised, particularly around lipolysis and changes in insulin sensitivity, the impact upon central processes, including that of the arcuate nucleus (ARC), are less well-defined in the setting of developmental programming. Early changes in the development of the GH-IGF neuroendocrine axis can modify the life course trajectory in mammalian species, and it is increasingly evident that GH modifies numerous aspects of hypothalamic function via hypothalamic GH receptors [49]. Developmental programming is commonly characterised by an increased appetite (hyperphagia) and is concomitant with hyperleptinemia in offspring, with leptin proposed to be the messenger by which the adipose tissue can influence hypothalamic regulation of GH secretion. However, surprisingly little has been studied on the effect of GH treatment on outcomes related to neuroendocrine programming, particularly given the feedback loops that exist between GH and key neurotrophic factors in early life, including leptin [50,51,52]. Of note, the somatotrope-specific deletion of the leptin receptor (OBRb) results in severe GHD and obesity in a sex-specific manner [53]. GH treatment to adult male rat offspring following maternal UN leads to differential effects on food intake (GH stimulated intake in controls but no response in hyperphagic programmed offspring), but there were no observed effects of GH treatment on circulating leptin concentrations [31]. Conversely, IGF-1 treatment to adult female offspring resulted in a normalisation of appetite and leptin concentrations [35]. However, no analysis of central pathways that may have mediated these altered responses was assessed.

It has been suggested that the extended elevation of GH can disrupt the normal physiological response to hyperleptinemia [54], thus predisposing towards leptin resistance and obesity, with the most likely mechanism underpinning leptin signaling impairment being at the level of the ARC. It has been well-established that adverse developmental programming disrupts the normal wiring of the pathways that regulate energy balance via a disruption in neurite outgrowth in the ARC and changes in the expression of anorixigenic/orexigenic factors, including neuropeptide Y (NPY), proopiomelanocortin (POMC), and agouti-related peptide (AgRP). Similarly, it is well-known that the developmental settings of the somatotropic neuroendocrine axis are highly sensitive to alterations in early postnatal nutrition, suggesting that this subset of hypothalamic neurons (NPY, POMC, and AgRP) are particularly sensitive to changes in nutrition during the perinatal period [55]. However, the interactions between the factors regulating energy balance and GH-releasing hormone (GHRH) neurons in the ARC in the setting of early life programming remain poorly defined, and may reflect the “adaptive plasticity” of somatotropic functions allowing individuals to modify (i.e., decelerate) growth and conserve energy resources as per the PARS hypothesis, and thereby improving fitness in challenging postnatal environments [56]. In cases of UN during lactation in the mouse, offspring show decreased circulating IGF-1 concentrations, which are associated with a reduced innervation of the median eminence by GHRH axons compared to control pups in the early neonatal period [55]. In vitro, IGF-1 stimulation preferentially stimulates axon elongation of GHRH neurons from normally nourished pups, whereas GHRH neurons derived from pups of UN mothers failed to respond to IGF-1 stimulation [55]. These effects appeared to be cell type-specific, as other ARC neurons, including AgRP, did not respond to IGF stimulation.These data highlight a cell-selective role for IGF-1 in axon elongation, and as part of the complex cellular mechanism that links UN during the early postnatal period with later programming of body growth trajectory.

The programming of growth is also associated with the IGF-1 receptor (IGF-1R) signaling pathway, with developing ARC neurons appearing to be highly sensitive to IGF-1 signaling, with the medio-basal hypothalamus being highly enriched for the IGF-1R [55,57]. As an example, the growth restriction arising from lactational caloric restriction can be mimicked by the tissue-specific inactivation of the IGF-1R in the central nervous system (CNS) [56]. In these studies, the selective inhibition of GH and IGF-1 pathways following birth was observed as a consequence of the partial inactivation of IGF-1R in the embryonic brain. This manifests as growth retardation, reduced adult body mass, and metabolic alterations that were favorably reflected in increased longevity [56]. Transient treatment with hGH in post-weaning offspring following maternal low protein (LP)-induced growth restriction did not prevent elevations in blood pressure in later life [58]. Conversely, pre-weaning bGH treatment to rat offspring exposed to global maternal UN results in the prevention of programming-induced hypertension and endothelial dysfunction in later life [45]. These differences in outcomes may reflect the type of GH used, the timing and window of treatment, the nature of the maternal dietary intervention, and the interactions therein.

It is well-established that maternal UN leads to increased adiposity in offspring, which is reflected in adipocyte hypertrophy. Pre-weaning GH treatment to the offspring of UN mothers normalises this hypertrophic phenotype, and thus may confer protection against lipid-induced metabolic dysfunction in later life [59]. Primary adipocytes derived from UN offspring displayed a significant increase in the secretion of pro-inflammatory cytokines, paralleled by an increase in the expression of cytokines/cytokine receptors. This correlated with increased toll-like receptor (TLR)-4/nuclear factor (NF)-κB signaling. While increased inflammatory potential was not observed in the adipose tissue derived from UN offspring treated with GH, alterations in the expression of genes relating to lipid and carbohydrate metabolism, along with nutrient transporters, were clearly evident [60]. There is also evidence that early GH treatment may confer protection in offspring against the so-called “second hit” in postnatal life. It is well-established that programmed offspring have heightened sensitivity to postnatal environmental triggers, such as exposure to an obesogenic environment, which markedly exacerbates the programmed phenotype [61]. As an example, maternal UN has been shown to prime hematopoietic immune cells in adult male offspring to a more potent pro-inflammatory phenotype, with heightened cytokine secretion and receptor expression. The stimulation of bone marrow macrophages using lipopolysaccharide resulted in a markedly increased secretion and expression of a range of pro-inflammatory markers in UN offspring [33]. Pre-weaning GH treatment of UN offspring prevented the appearance of this pro-inflammatory phenotype.

**Figure 1:**
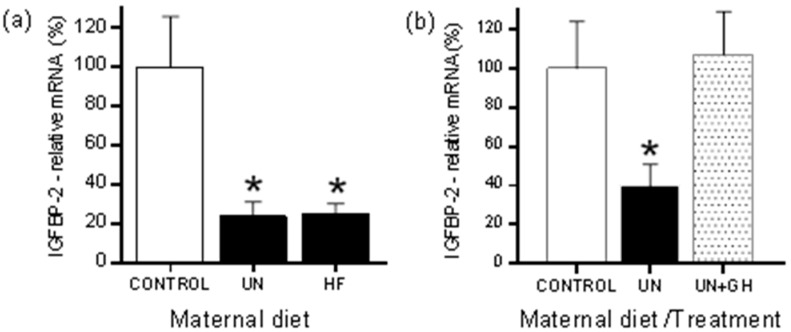
Hepatic insulin-like growth factor binding protein 2 (IGFBP-2) expression in (**a**) adult male offspring from mothers either undernourished (UN) or fed high fat (HF) diets during pregnancy as compared to offspring from normally nourished mothers; (**b**) hepatic IGFBP-2 expression in adult male offspring of UN mothers treated with growth hormone (GH) during the pre-weaning period. * *p* < 0.05 versus Control. Data are mean ± standard error of the mean (SEM), *n* = 10 per group. Note that the UN groups shown for (**a**,**b**) are derived from independent animal cohorts. Figure 1a reimaged from [63].

Although the efficiency of GH and IGF-1 as a treatment paradigm in the setting of maternal UN has been reasonably well explored in the rodent, less is known about the effect of such interventions in the setting of maternal overnutrition. Work by Dunn and Bale in the mouse highlighted the potential for maternal obesity to exert transgenerational effects on the GH-IGF axis via persistent changes in circulating IGF-1/IGFBP-3 concentrations across multiple generations [62]. Although the phenotypic outcomes in offspring are similar across both models, as characterised by rapid catch-up growth, increased adiposity, and cardiometabolic dysregulation, whether the mechanisms are similar is not well defined. Both maternal UN and maternal high fat nutrition can lead to similar effects on components of the IGF-IGFBP system in adult offspring, including an altered hepatic expression of IGFBP-1 and IGFBP-2 (Figure 1a) [63]. However, it must be noted that a maternal high fat (HF) dietary intake can also represent a form of malnutrition due to macronutrient deficiencies, so that may account for some of the similarities observed across seemingly disparate nutritional models.

### 2.2. Sheep

A number of studies have been undertaken in the sheep to examine the effects of early GH administration on maternal and fetal outcomes. Some of this work has been reviewed previously [64]. Periconceptional GH treatment has been reported to alter fetal growth and development in lambs, with persistent changes in the GH axis into the postnatal period, including increased body weights and a lack of responsiveness in IGF-1 to a GHRH challenge [65]. Similar effects were observed in a further similar study with increased lamb weights in those born to normal ewes treated with GH at the time of breeding, possibly via increased placental efficiency and increased birth weights [66].

Chronic maternal GH administration has been shown to increase placental transport capacity, direct maternal effects of GH treatment may result in in a limited fetal substrate supply, and thus prevent increased fetal growth [67]. Similarly, GH (by infusion) to the normally growing late-gestation fetal sheep does not affect fetal growth nor metabolism [68]. Using a similar experimental paradigm in growth-restricted fetal sheep, chronic pulsatile GH treatment restored fetal IGF-1 concentrations, but did not impact on fetal growth and indeed further reduced intestinal and renal weights [69]; these changes in fetal IGF-1 concentrations may reflect reduced clearance rather than increased production [70]. Such results may not be unexpected given the early work by Curran et al., where data suggested that pregnancy in states of GH-deficiency is not detrimental to the mother or fetus [71]. Moreover, in the fetus, GH plays little or no role in the regulation of fetal growth, and the IGFs, particularly IGF-2 [72], control growth directly and independently of fetal GH secretion. In an overnourished sheep model, GH treatment in later pregnancy led to an increase in fetal weights reflected by increased adipogenesis, without effects on fetal leptin concentrations, possibly indicative of a direct effect of GH on adipose in utero [73]. Of note, during human pregnancy, maternal pituitary GH (GH-N) concentrations are suppressed, and the placental variant GH (GH-V) becomes the predominant GH in the mother. Importantly, pregnancy-induced increases in GH-V in GHD mothers are comparable to the rise seen during normal pregnancies, and are not suppressed by concurrent GH treatment [74]. However, non-primate animals do not have a placental variant of GH.

Data derived from sheep have been variable, and depend on the timing and mode of GH treatment. In a model of IUGR induced by placental embolization, GH treatment increased fetal growth rates and fat accrual, but was associated with hydraencephalic brain lesions in some fetuses [75]. Thus, although GH treatment either to mother or fetus in the sheep had variable impact and was possibly detrimental to offspring outcome, more positive effects have been shown in these models using fetal intra-amniotic IGF-1 treatment [75,76], with improved growth rates following IUGR independent of changes in fetal plasma IGF-1 or insulin concentrations. As an example, intra-amniotic IGF-1 treatment given weekly increased the growth of IUGR fetuses via increases in the fetal substrate supply, upregulation of amino acid transporters in the placenta, and changes in the mTOR pathway [77]. These studies suggest that amniotic IGF-1 treatment may provide the basis for a clinically applicable prenatal treatment for the IUGR fetus [76]. As above, in the overnourished adolescent sheep, late maternal GH treatment can increase fetal adiposity [73]. In this model, nutrient partitioning favors maternal growth at the expense of placental and subsequently fetal growth. Treatment with GH during late gestation (d95 to d125) can alter nutrient partitioning to increase the endogenous maternal and fetal glycemia that persists for the duration of treatment. Such treatment, given peak fetal nutrient demand during this period, can enhance fetal growth and modestly ameliorate the growth restriction observed in untreated animals. However, this in utero catch-up growth is associated with a significant increase in fetal fat mass [73]. Increased fetal adiposity was not observed in a further study by the same group examining GH treatment at earlier gestational time points (d35 to d80), where altered nutrient partitioning was also observed in favor of uteroplacental and fetal growth, although GH treatment was associated with polyhydramnios [78].

### 2.3. Other Models

Although the primary model used in the rodent is around global or targeted nutrition deficits, some work has also been undertaken in models of feto-placental insufficiency. In a rat model of IUGR using uterine artery ligation, GH treatment resulted in body weight recovery in offspring in a sex-specific manner, with recovery being more rapid in females compared to males [79].

In a further rat model of uterine artery ligation, IGF-1 treatment during the early postnatal period saw no effect on body or brain weight, which was associated with an increase in hepatic IGFBP-3 [80]. Little work has been undertaken in the non-human primate (NHP) model in the context of developmental programming. Limited data in normal rhesus monkeys suggest that neonatal body weight gain is accelerated in nursing infants whose mothers have received GH from at least the second trimester of pregnancy through to the period of lactation [81].

## 3. Potential Role of Epigenetics

Increasing evidence supports a role for epigenetic mechanisms in understanding how early life factors, including poor nutrition during pregnancy and infancy, influence growth and metabolic function in postnatal life. The treatment of short children using GH to optimise growth is characterised by variable efficacy; the causes of this individual variability are multifactorial, with recent work showing that, in addition to a polymorphism in the GH receptor arising due to the common deletion of exon 3, epigenetic processes may be a major determinant of an individual’s responsiveness to GH treatment [82]. As an example, Ouni et al. have shown that altered methylation of the proximal part of the IGF-1 P2 promoter is associated with individual variability in circulating IGF-1 and height in growing children [83]. In a model of programming induced via maternal low salt intake, low birth weight is correlated with IGF-1 DNA methylation in neonates [84].

The impact of promoter DNA methylation on the regulation of the GH gene is not well-described [85]. Several studies have now identified methylation as a regulatory component for GH gene expression [86,87,88], with work in the rat implicating site-specific methylation as modulating the action of transcription factors that dictate the tissue-specific expression of the GH gene in vivo [86]. GH promoter regulation is via both positive and negative DNA elements through co-regulatory proteins and transcription factors [89]. DNA methylation near the transcriptional start site is associated with GH gene expression, and site-specific methylation may therefore mediate the action of transcription factors that dictate the tissue-specific expression of GH [86]. In the mouse, genomic DNA from the pituitary is hypomethylated over the GH promoter region [85]. In the setting of developmental programming, little has been reported on the role of epigenetic regulation of GH itself. However, there have been a number of reports on epigenetic changes in pathways associated with components of the GH axis, including adverse in utero environments linked to altered DNA methylation at the IGF-2/H19 locus [90]. IGF-2 is transcribed in most tissues only from the paternal chromosome, while H19 is transcribed only from the maternal allele, with the allelic methylation patterns of each gene arising early in embryogenesis and changing progressively during development [91]. It has been hypothesised that the maternal and paternal expression patterns are balanced to offer maximal benefit to fetal growth whilst preventing excessive depletion of the mother’s resources [92]. Although not under GH control, the IGF-2/H19 locus, as part of the IGF axis, is the most characterised of all identified imprinted genetic loci, and has been implicated in adverse development programming via altered DNA methylation in both rodents [93] and humans [90,94]. Parent of origin imprinting disorders mediated by aberrant DNA methylation in the IGF-2/H19 region are associated with distinct alterations in growth phenotypes [90,95]. In the rat, maternal LP dietary intake alters hepatic IGF-2 and H19 expression via alterations in the DNA methylation of these genes [93]. A maternal LP diet results in the hypermethylation of the imprinting control region of the IGF-2/H19 locus and changes in the DNA methyltransferase family (LP-induced increases in Dnmt1 and Dnmt3a), but this can be prevented with maternal folic acid supplementation [93]. In the Dutch Famine cohort, offspring exposed to maternal famine in early gestation had reduced methylation at the IGF-2 differentially methylated region (DMR), an imprinted region of differential methylation within the IGF-2 gene [94], and were associated with obesity in later life [96,97].

A further area of interest is that of IGFBP-2, a leptin-regulated gene [98]. Both maternal UN and overnutrition have been shown to lead to a reduction in hepatic IGFBP-2 expression (Figure 1a) [63]. In work by Smith et al. [63], either maternal UN or high fat nutrition leads to an offspring phenotype characterised by increased adiposity, insulin and leptin resistance, and alterations in blood lipids in offspring as compared to control born to normally nourished mothers. The circulating IGF-1 and IGFBP-3 concentrations and hepatic IGFBP-1 and IGFBP-2 expression were significantly decreased in both dietary groups compared to controls. The promoter region of IGFBP-2 is CpG rich and is highly conserved in mammalian species [99], and therefore it is plausible that alterations in methylation play an important role in the mediation of IGFBP-2 gene expression. However, despite the observed marked reduction in hepatic IGFBP-2 mRNA expression across both UN and high fat animal models, Smith et al. observed no significant differences in IGFBP-2 methylation, with a generalised hypomethylation of the IGFBP-2 promoter across all dietary groups [63]. Similar patterns of IGFBP-2 hypomethylation have been described in other experimental species [100]. Of note, neonatal GH treatment can reverse the programming-induced reduction in hepatic IGFBP-2 expression, and restore expression to that of offspring of normally nourished mothers (Figure 1b).

Although DNA methylation is the most widely studied of the epigenetic readouts, there is also evidence for GH-mediated changes in microRNAs (miRNAs) arising due to developmental programming. In a rodent model of maternal UN, pre-weaning GH treatment has been shown to reverse programming-induced hypertension and associated cardiac hypertrophy in offspring [46]. This appears to be mediated, at least in part, by the upregulation of a group of miRNAs, particularly the LET-7 family, that are known to be associated with inflammation and cardiovascular development [46,101].

## 4. Discussion

Epidemiological studies in humans and an extensive array of animal models have clearly shown that an altered early life environment, particularly poor nutrition, leads to an increased risk for a range of cardiometabolic and behavioral diseases in later life. Alterations in the GH-IGF axis play a major role in the metabolic derangements induced in offspring following adverse developmental programming. The phenotypes that manifest as a consequence of aberrant developmental programming are similar to that seen in cases of GHD. A number of studies have now shown that, under conditions of adequate postnatal nutrition, both GH and IGF-1 can equally promote postnatal catch-up growth in rats with IUGR arising due to suboptimal maternal nutrition. In addition, experimental work in the rodent suggests that disparate models of altered early life nutrition that elicit similar growth and cardiometabolic dysregulation in offspring may be associated with common alterations in the GH-IGF-IGFBP pathway [63].

Although epidemiological studies have shown persistent changes in the GH-IGF axis in offspring arising from, for example, famine exposure, little is known regarding the GH-IGF axis at the time of the exposure. Data on efficacy and long-term effects drawn from clinical studies of childhood or adult GHD are also limited due to the low prevalence of treatment cases relative to cases of short stature and IUGR [102]. Although some initial issues were raised around the side effects of GH treatment in the clinic, including increased risk for T2DM, the longer term follow-up studies would indicate that GH treatment is safe in the longer term, and the initial side effects observed may relate to an initial trade-off for longer term benefits. Less is known around IGF-1 as a treatment modality due to some early concerns raised around cancer risk [103], although such linkage remains controversial [104,105]. IGF-1 treatment in programmed adult rat offspring ameliorates most of the adverse outcomes, but longer term effects or effects of early intervention have not been well-described.

Treatments with both neonatal leptin and pre-weaning GH have shown marked efficacy in ameliorating a range of programmed disorders, further reinforcing this early life period as a critical period for intervention. It has been suggested that early life leptin treatment leads to the normalisation of cues around the nutritional environment the offspring are exposed to; i.e., sending signals of “good nutrition”. Similar effects may be initiated via GH or IGF treatment to thus curb developmental mismatch and maladaptive PARs. This was highlighted in the pre-weaning GH treatment study, whereby the priming of secondary environmental triggers that would normally exacerbate the UN phenotype was prevented in postnatal life in those offspring treated with GH in early life. Indeed, the effects of GH in the early life period may be mediated through effects on the leptin axis. However, the crosstalk relationship between the GH-IGF axis and leptin remains poorly defined. The overexpression of leptin in transgenic mice results in decreased circulating IGF-1 concentrations, thus suggesting that leptin could be a primary regulator of IGF-1 secretion [106]. Clinically, it has also been reported that GH and IGF-1 can influence circulating leptin concentrations, albeit in patients with end-stage kidney disease [107].

Some lack of consistency in the experimental literature primarily reflects either differences in the GH preparation used, e.g., hGH versus bGH, timing of exposure, mode of delivery (injection versus infusion) or sex-specific effects. In addition, the cases of nutritional deficit models may vary dependent upon the severity of the UN and the nature of the dietary restriction (e.g., global UN, low protein). Further, as shown with other intervention strategies in programming models, the effects observed may be directionally dependent upon the prior nutritional status of the mother [63], and in some cases treatment may be causal in aberrant phenotype development when given to offspring of normally nourished mothers.

In summary, GH and components of the IGF-I system appear to be efficacious in ameliorating some of the outcomes resultant from aberrant developmental programming, but more work is required, particularly around potential epigenetic mechanisms (which remain largely associative in nature with limited evidence for direct causality from the data to date), transgenerational effects, and sexually dimorphic responses to treatment. Further, although the changes in the GH-IGF axis that manifest via either maternal UN or overnutrition are broadly similar across a range of animal models, it remains to be defined whether the mechanisms leading to the dysregulation of the pathways are the same, and that treatment interventions with GH or IGF-1 will have similar efficacy across models.
